# Development and validation of a clinical HPLC method for the quantification of hydroxychloroquine and its metabolites in whole blood

**DOI:** 10.4155/fso.15.24

**Published:** 2015-11-01

**Authors:** Ying Qu, Gaelle Noe, Autumn R Breaud, Michel Vidal, William A Clarke, Noel Zahr, Thierry Dervieux, Nathalie Costedoat-Chalumeau, Benoit Blanchet

**Affiliations:** 1 Research & Development, Exagen Diagnostic, 1261 Liberty Way, Vista, CA 92081, USA; 2 UF de Pharmacocinétique et Pharmacochimie, Hôpital Cochin, APHP, 27 Rue Faubourg St Jacques, 75014 Paris, France; 3 Clinical Chemistry, Department of Pathology, The John Hopkins University, Baltimore, MD 21287, USA; 4 UMR8638 CNRS, UFR de Pharmacie, Université Paris Descartes, PRES Sorbonne Paris Cité, Paris, France; 5 Laboratoire de Pharmacologie, Groupe Hospitalier Pitié-Salpêtrière, APHP, 47–83 Boulevard de l'Hôpital, 75651 Paris Cedex 13, France; 6 Université Paris-Descartes, Paris, France; 7 AP-HP, Hôpital Cochin, Centre de Référence Maladies Auto-Immunes et Systémiques Rares, Service de Médecine Interne, 27 Rue du Faubourg Saint-Jacques, 75014 Paris, France

**Keywords:** clinical drug monitoring, desethylchloroquine, desethylhydroxychloroquine, drug monitoring, fluorescence detection, HPLC, hydroxychloroquine, LC–MS/MS, systemic lupus erythematosus, whole blood

## Abstract

**Background:**

Therapeutic drug monitoring for hydroxychloroquine (HCQ) has been suggested to assess nonadherence and optimize treatment efficacy in systemic lupus erythematosus patients.

**Materials & methods:**

After protein precipitation, HCQ and its metabolites, desethylhydroxychloroquine and desethylchloroquine were separated on a phenyl column and monitored by fluorescence detection. The method was linear from 50 to 4000 ng/ml for HCQ. The intra-day and inter-day precision of HCQ, desethylhydroxychloroquine and desethylchloroquine ranged from 4.3 to 10.3%. LLOQ was 50 ng/ml for HCQ.

**Conclusion:**

The method is very practical and was applied to routinely monitor the steady state whole blood exposure of HCQ and its metabolites in systemic lupus erythematosus patients. It well correlated with our LC–MS/MS and another HPLC method.

## Abstract (lay abstract)

Plaquenil (hydroxychloroquine) is an inexpensive antimalarial drug, now regarded as a safe and reasonably effective treatment for various autoimmune rheumatic diseases including systemic lupus erythematosus. We developed a simple HPLC method for monitoring the hydroxychloroquine concentration in patient whole blood. The clinical indication for the testing is to identify patient nonadherence and optimize treatment efficacy. The method is easy to operate in a clinical laboratory. The result well correlated with other methods.

**Figure 1.**
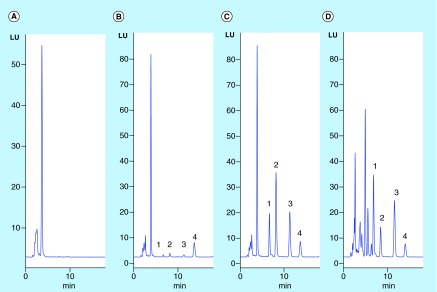
**Representative chromatograms.** **(A)** Blank human whole blood; **(B)** LLOQ of hydroxychloroquine (HCQ; 50 ng/ml), desethylhydroxychloroquine (DHCQ; 25 ng/ml) and desethylchloroquine (DCQ; 25 ng/ml) standard in human whole blood; **(C)** spiked HCQ (1000 ng/ml), DHCQ (500 ng/ml) and DCQ (500 ng/ml) standard in human whole blood; **(D)** whole blood sample from systemic lupus erythematosus patient treated with 400 mg/day of HCQ, the concentration of HCQ, DHCQ and DCQ are 1126 ng/ml, 894 ng/ml and 113 ng/ml, respectively. Identity of the peaks: 1) DHCQ; 2) DCQ; 3) HCQ; 4) Internal standard (IS, Quinine).

**Figure 2.**
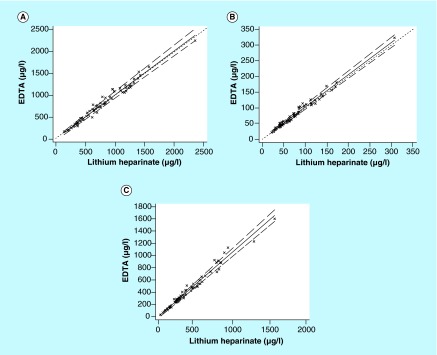
**Passing–Bablok regression analysis between the EDTA and lithium-heparinate tubes for (A) hydroxychloroquine, (B) desethylchloroquine and (C) desethylchloroquine assay.** A total of 65 whole blood samples were collected from systemic lupus erythematosus patients treated with hydroxychloroquine. The solid and dashed lines indicate the regression line and CI for the regression line, respectively. Whatever the analyte, no significant deviation from linearity was observed (p > 0.1). The formula of regression equations are the following: EDTA = 1.01 (95% CI: 0.96–1.05) × lithium-heparinate + 11.7 (95% CI: -19.0–39.8) for hydroxychloroquine. EDTA = 1.03 (95% CI: 0.98–1.07) × lithium-heparinate + 1.2 (95% CI: -2.0–3.8) for desethylchloroquine. EDTA = 1.06 (95% CI: 1.0–1.11) × lithium-heparinate + 1.1 (95% CI: -16.0–8.0) for desethylhydroxychloroquine.

**Figure 3.**
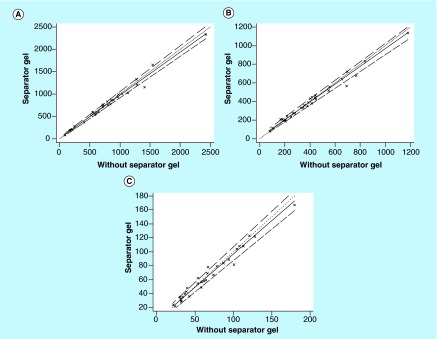
**Passing–Bablok regression analysis between lithium-heparinate tubes containing gel separator and those without for (A) hydroxychloroquine, (B) desethylchloroquine and (C) desethylhydroxychloroquine assay.** A total of 37 whole blood samples were collected from systemic lupus erythematosus patients treated with hydroxychloroquine. The solid and dashed lines indicate the regression line and CI for the regression line, respectively. Whatever the analyte, no significant deviation from linearity was observed (p > 0.1). The formula of regression equations are the following: Tubes with gel separator = 0.96 (95% CI: 0.93–1.02) × tubes without gel separator + 11.7 (95% CI: -12.5–15.8) for hydroxychloroquine. Tubes with gel separator = 0.95 (95% CI: 0.90–1.02) × tubes without gel separator + 1.39 (95% CI: -2.76–5.19) for desethylchloroquine. Tubes with gel separator = 0.95 (95% CI: 0.90–1.00) × Tubes without gel separator + 14.0 (95% CI: 2.0–34.9) for desethylhydroxychloroquine.

**Figure 4.**
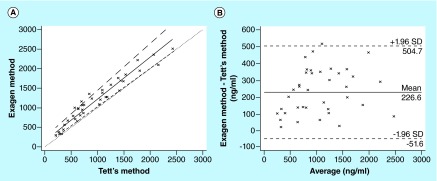
**Analysis of patient sample with current method and Tett's method.** **(A)** Passing–Bablok regression analysis between the new method and the Tett's method for 37 whole blood samples from systemic lupus erythematosus patients treated with hydroxychloroquine. The solid and dashed lines indicate the regression line and CI for the regression line, respectively. **(B)** Bland–Altman analysis between the new method and the Tett's method for 37 whole blood samples from systemic lupus erythematosus patients treated with hydroxychloroquine. The solid line indicates the mean difference between the methods.

**Figure 5.**
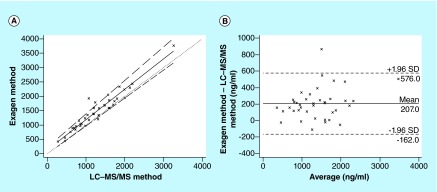
**Analysis of patient sample with current method and LC–MS/MS method.** **(A)** Passing–Bablok regression analysis between the new method and the LC–MS/MS method for 37 whole blood samples from systemic lupus erythematosus patients treated with hydroxychloroquine. The solid and dashed lines indicate the regression line and CI for the regression line, respectively. **(B)** Bland–Altman analysis between the new method and the LC–MS/MS method for 37 whole blood samples from systemic lupus erythematosus patients treated with hydroxychloroquine. The solid line indicates the mean difference between the methods.

Hydroxychloroquine (HCQ) is an antimalarial drug effective in the treatment of various autoimmune rheumatic diseases including systemic lupus erythematosus (SLE) and rheumatoid arthritis (RA). HCQ is currently the most widely prescribed drug in SLE and RA. In SLE patients, HCQ can help prevent the emergence of disease flares [1] while also minimizing the risk for developing diabetes mellitus, thrombosis and organ or overall damage accrual [2–5]. It may therefore improve survival in SLE [6–8].

HCQ is almost completely and rapidly absorbed after oral administration. About 50% of the HCQ in plasma is bound to proteins. HCQ is metabolized in the liver through CYP450 isoenzyme 2D6 into two major oxidative metabolites, desethylhydroxychloroquine (Cletoquine; DHCQ) and desethylchloroquine (DCQ) [9]. Only DHCQ is an active metabolite [10]. Besides, Brocks *et al*. showed that HCQ concentrations in plasma were appreciably lower and more variable than those in whole blood, which suggests that whole blood would be the optimal matrix to use for therapeutic drug monitoring (TDM) of HCQ [11]. Finally, Somer *et al*. documented a large inter-patient variability in whole blood concentration of HCQ (˜11-fold) [9].

Several studies have reported a pharmacokinetic/pharmacodynamic relationship for HCQ in patients with SLE and RA [12,13]. In SLE patients, low whole blood HCQ concentrations were associated with ongoing disease activity and is a marker and predictor of SLE flares [13]. Additionally, whole blood HCQ concentrations can be used to identify nonadherence of SLE patients [14]. Therefore, TDM for HCQ might help to both identify nonadherence and SLE patients who are at risk of disease exacerbation, and therefore optimize treatment efficacy. Although whole blood DHCQ concentration was identified as a significant predictor for efficacy in patients with active RA [12], the benefit of its monitoring has not been extensively investigated in SLE patients. Up to now, the most popular method to assay whole blood HCQ concentrations in clinical studies is HPLC coupled to the fluorescence detection (HPLC-FLD). Particularly the Tett's method with different modifications has been used in many clinical reports [13,15–18]. However, these methods have the disadvantage of being labor intensive and causing laboratory pollution related to the liquid–liquid extraction. In recent years, LC–MS/MS methods have been developed and validated for quantification of HCQ in whole blood [19–21]. However, such equipment is not available in all laboratories, which limits the spreading of TDM for HCQ in daily clinical practice. The aim of this work was to develop and validate a simple, precise and sensitive HPLC-FLD method to quantify HCQ and its metabolites (DCQ, DHCQ) in whole blood from SLE patients. Additionally, the interchangeability between this new method and the reference methods (Tett's method and LC–MS/MS) [15,20] for HCQ assay was assessed in blind fashion between three clinical laboratories.

## Materials & methods

### Chemicals

Hydroxychloroquine (HCQ; 7-chloro-4-[4-(N-ethyl-N-b-hydroxyethylamino)-1-methylbutylamino]quinoline sulfate) was purchased from VWR (CA, USA) which is a US Pharmacopeia (USP) Reference Standard and made by USP Convention. Cletoquine oxalate (DHCQ) and DCQ were purchased from Toronto Research Chemicals (Toronto, Canada). Quinine hemisulfate salt monohydrate (QN) was used as the internal standard (IS) and purchased from Sigma-Aldrich (MO, USA). The common background drugs prescribed for SLE and rheumatoid arthritis (RA) patients, such as azathioprine (AZA), 6-tioguanine (6-TG), mycophenolic acid (MYCO), chloroquine (CQ), prednisone, celecoxib were purchased from Sigma-Aldrich and methotrexate (MTX) was from VWR. HPLC grade water were purified by Pure Lab Ultra system (ELGA LLC, IL, USA) at Exagen. Methanol, glycine and cupric sulfate were purchased from Sigma-Aldrich. Sodium chloride was purchased from VWR.

### Preparation of standard stock solutions

HCQ and DHCQ were prepared in water. QN and DCQ were prepared in 50% methanol in water. After dissolution, the final concentration was confirmed by using a DU730 Life Science UV/Vis Spectrophotometer (Beckman Coulter, CA, USA) and the molar extinction coefficients of HCQ (ϵ_329nm_ = 17,000), DHCQ (ϵ_329nm_ = 17,000), DCQ (ϵ_330nm_ = 18,000) and QN (ϵ_330nm_ = 4700). The HCQ, DHCQ and DCQ standards were diluted to a final concentration of 200 μg/ml as standard stock solution and IS was diluted to a final concentration of 1 mg/ml in water. All standard and IS stock solutions were stored at -80°C. An independent solution from a different lot of HCQ was also prepared as described above, and then used as control stock solution for the HPLC assay.

### Chromatographic apparatus & conditions

The liquid chromatograph was an Agilent 1200 HPLC system consisting of a binary pump, a mobile phase degasser, a thermostatic column compartment, an auto-sampler (kept at 4°C) and a fluorescence detector. Agilent ChemStation software was used to control the system and analyze the data. The chromatographic separation was performed on XTerra phenyl^®^ column (250 × 4.6 mm, 5 μm; Waters, MA, USA) associated with guard column packed with the same bonded phase. XTerra^®^ Columns combine the properties of silica and polymeric bonded phases with patented Hybrid Particle which enables chromatographers to perform high pH method development. The temperature of column was controlled at 50°C. The mobile phase consisted of a mixture of glycine buffer/sodium chloride (pH 9.7, 100 mM) and methanol (46:54; v/v). It was delivered at a flow rate of 1.2 ml/min throughout the 18-min run. The analytes were monitored by fluorescence detection with excitation wavelength at 320 nm and emission wavelength at 380 nm.

### Standard curve & control preparation

Working standards of 200, 100, 50, 25, 12.5, 2.5 µg/ml for HCQ; 200, 50, 25, 12.5, 6.25, 1.25 µg/ml for DHCQ; 100, 50, 25, 12.5, 6.25, 1.25 µg/ml for DCQ and 50 µg/ml for QN (IS) were freshly prepared in water on the day of experiment. Calibration standards of 4000, 2000, 1000, 500, 250, 50 ng/ml for HCQ; 2000, 1000, 500, 250, 125, 25 ng/ml for DHCQ; 1000, 500, 250, 125, 25 ng/ml for DCQ and 5000 ng/ml for QN were freshly prepared in HCQ-free human blood on the day of experiment as well. The large concentration range of HCQ standard curve was chosen related to the whole blood HCQ concentrations previously reported in the literature [14]. Calibration standards were prepared by spiking human HCQ-free whole blood with appropriate working solutions. Concentrations of control were 250, 1000 and 2000 ng/ml for HCQ; 125, 500 and 2000 ng/ml for DHCQ and 125, 500 and 1000 ng/ml for DCQ. Controls were prepared in a similar way by using control stock solution.

### Sample preparation

First, 200 µl of whole blood (standard, control and patient sample) was distributed into 1 ml Eppendorf tube; then 20 µl of IS at 50 µg/ml was added and mixed well by vortex for 5 s. A protein precipitation mixture was prepared by spiking 400 µl cold methanol (stored at -20°C before use) and 50 µl cupric sulfate (3 mM) to the samples. After a 2 min vortexation step on a Multi-Tube Vortexer (VWR) at speed 10, samples were centrifuged for 10 min at 14,000 rpm. The supernatant was transferred into a polypropylene sample vial. The volume of 50 μl of supernatant was directly injected into the HPLC system.

### Specificity & selectivity

Assay selectivity was achieved by fully separating HCQ from its two major metabolites, DHCQ, DCQ and other unknown peaks. Specificity was evaluated by direct HPLC injection of standard solution (1000 ng/ml) of a number of common background drugs, especially those used in the treatment of SLE patients, such as azathioprine, 6-tioguanine, mycophenolic acid, chloroquine, prednisone, celecoxib and methotrexate. Finally, interferences with endogenous compounds have been evaluated from whole blood samples from HCQ-naive patients experiencing severe hepatic (n = 5) or renal (n = 5) impairment.

### Method validation

The method was initially developed in Cochin hospital, optimized in Exagen and validated in all four laboratories. The method was validated according to the US FDA guidelines for bioanalytical method validation [22].

Linearity of the method was determined by replicate analysis of six complete standard curves on six separate days. HCQ, DCQ and DHCQ concentrations of unknown blood samples were back-calculated from a linear standard curve of peak area ratio of HCQ to the IS versus their concentration ratio. The linear standard curve was equal weight and force origin from zero. The three levels of control were assayed to validate each standard curve.

Intra- and interday precision (relative standard deviation of the mean [RSD%]) and accuracy for each analyte (HQC, DCQ, DHCQ) were evaluated at the three levels of control. Ten replicates of each level were assayed in one run for the intra-day experiment. Four replicates of each level were assayed within 5 different days for the interday experiment. According to FDA guidelines, the accuracy and precision for all tested concentrations should be within ±15%.

The LOD was determined from the smallest measurable peaks which signal to noise ratio equals to 3. The LLOQ was defined as the lowest concentration of analyte (HCQ, DCQ and DHCQ) for which accuracy and precision should not exceed 20%.

Carryover was evaluated by injecting a 2000 ng/ml concentration sample (HCQ, DCQ and DHCQ) followed an injection of blank whole blood sample. The analyte concentration of blank was accepted whether it was found to be less than LOD.

Average recovery of HCQ and its metabolites was determined by comparing pre- and post-extraction samples of four levels of control for each analyte (HCQ, DCQ and DHCQ). Three sets pre-extraction samples at the concentration of 50, 250, 1000, 2000 ng/ml for HCQ, DHCQ and DCQ, were prepared by spiking standard in whole blood. Three sets post-extraction samples at each concentration were prepared by first extracting blank whole blood and subsequently spiking the supernatants with HCQ, DHCQ and DCQ standard to give a final concentration equivalent to that obtained for the pre-extraction samples. The extraction efficiency was calculated according to the following equation:Equation 1
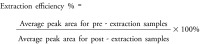



The stock solutions of HCQ, DHCQ, DCQ and IS stored in the dark at -80°C were compared monthly to a stock solution freshly made during a 12-month period. The acceptance criterion was ±15% difference between peak areas of both solutions for each compound. The stability of HCQ and its metabolites was assessed using the three levels of control (in triplicate for each) for freeze–thaw, short-term stability and post-preparative stability. Briefly, the freeze–thaw samples underwent three cycles of freeze and thaw before preparing the samples for analysis. For the assessment of short term stability, samples were kept in dark for 7 days at room temperature or at 4°C before extraction. Finally, post-preparative stability was evaluated by keeping the extracted samples in the autosampler at 4°C for 48 h. For all these experiments, stability was acceptable when ≥85% of the analyte was recovered.

### Blood collection

To investigate the effect of anticoagulant on HCQ assay, whole blood samples from 65 SLE patients treated with HCQ were collected in both EDTA and lithium-heparinate tubes (Becton Dickinson, NJ, USA) without gel separator. In 37 of these 65 patients, an additional whole blood sample was collected in a lithium-heparinate tube containing gel separator. All patients agreed for the sampling analysis in compliance with French regulations.

### Clinical application

Whole blood from 37 SLE patients treated with HCQ therapy (daily dosing range 200–600 mg/day) was collected at steady-state in 5 ml EDTA tubes, then aliquoted in polypropylene tubes and stored at -20°C until analysis. For the establishment of the interchangeability between current method and the reference methods (Tett's method, LC–MS/MS) [15,20] for HCQ assay, three clinical laboratories were involved: Centre Hospitalier Universitaire Pitié-Salpêtrière (Paris, France), Exagen Diagnostic (Vista, USA) and John Hopkins Hospital (Baltimore, USA). An aliquot of frozen whole blood was sent in a blinded fashion to assay whole blood HCQ concentration in each clinical laboratory. Tett's method analysis was performed in Centre Hospitalier Universitaire Pitié-Salpêtrière, Current HPLC-FLD method was applied in Exagen Diagnostic and LC–MS/MS method was developed and used in John Hopkins Hospital [15,20].

### Statistical analysis

The nonparametric regression proposed by Passing–Bablock *et al*. was used firstly to investigate the anticoagulant and gel separator effect on assay of HCQ and its metabolites, and then to estimate the relationship between two analytical techniques to assay whole blood HCQ concentration [23]. The regression equation is expressed with the 95% CI for the estimates of slope and intercept. The scatter of the method was visualized according to the recommendations of Bland and Altman [24]. Statistical analysis was performed using Medcalc^®^ software, version 7.3.0.1 (Mariakerke, Belgique) after exclusion of the samples below the LLQ of either method. p < 0.05 was considered statistically significant.

## Results & discussion

### Selectivity & specificity

Representative chromatograms are depicted in Figure 1, showing blank whole blood; LLOQ of standard in whole blood; high concentration standard in whole blood; and whole blood sample from SLE patient treated with HCQ. No significant interference from endogenous or exogenous compounds was observed in the chromatograms of whole blood from ten patients experiencing severe renal or hepatic impairment. Direct injection of standard solution (1000 ng/ml) of common background drugs used for SLE and RA treatment, AZA, 6-TG, MYCO, CQ, Prednisone, Celecoxib and MTX, did not lead to interfering peaks at the retention times of HCQ, DCQ, DHCQ and quinine. Finally, there was no significant carry-over following an injection of 2000 ng/ml HCQ, DCQ and DHCQ.

### Linearity, accuracy, precision & sensitivity

Standard curve demonstrated a linear relationship between peak area ratio of analyte (HCQ, DCQ and DHCQ) to the IS versus their concentration ratio with mean correlation coefficients >0.995 (Table 1). The analytical method demonstrated the linearity range is from 50 to 4000 ng/ml for HCQ, 25 to 2000 ng/ml for DHCQ and 25 to 1000 ng/ml for DCQ. The intraday and interday validation data for three levels of control are presented in the Table 2. The intraday and interday accuracy and precision were well within 15%. The values for intraday and interday accuracies of HCQ, DHCQ and DCQ ranged from 93.1 to 103.2%, with precision ranged from 4.3 to 10.3%. The values of accuracy and precision for HCQ, DHCQ and DCQ prove both the reliability and the reproducibility of the method. The LLOQ for HCQ (50 ng/ml) is approximately threefold higher than that previously reported with the LC–MS/MS method [20]. In the present study, the mean steady-state HCQ concentration was 1336 ± 621 ng/ml in 37 SLE patients treated with HCQ dose ranging from 200 to 600 mg/day, which suggests that our LLOQ is clinically relevant for TDM of HCQ. The LLOQ for DHCQ and DCQ (25 ng/ml) is similar with recent LLOQ reported with LC–MS/MS [21]. Additionally, the mean steady-state DHCQ and DCQ concentration was 1037 ± 721 ng/ml and 115 ± 54 ng/ml, respectively. Together, these results indicate that the LLOQs for the two metabolites are also clinically relevant for TDM. Finally, LOD was 25 ng/ml for HCQ and 12.5 ng/ml for DHCQ and DCQ.

### Recovery

The recovery results for different compounds are presented in Table 3. All the percentage recoveries were acceptable (˜55%) with a good reproducibility of the extraction for all compounds (RSD% less or equal to 15%). The mean recovery of quinine (IS) was 62.4 ± 2.5%. Besides, the absolute recovery of HCQ and its metabolites was sufficient to achieve sensitivity required for TDM in whole blood from SLE patients. Other protein precipitation method, such as percholric acid, has been tested and cannot be used in this FLD detection since HCQ is detectable with high emission intensity of fluorescence at high pH. The recovery was optimized by testing different concentration of cupric sulfate. Overall, the present simple pretreatment procedure using a low volume of whole blood sample (200 µl) is selective, reproducible and rapid.

### Stability

The stock solutions of HCQ, DHCQ, DCQ and IS stored for 12 months at -80°C were comparable to the stock solutions freshly made. The results of the stability test that includes freeze-thaw, short-term and auto-sampler stability are summarized in Table 4. Whole blood HCQ, DHCQ and DCQ concentrations were unchanged when stored in the dark, at ambient temperature or at 4°C for 7 days. Freeze-thaw stability experiment showed that HCQ, DHCQ and DCQ in human whole blood were stable for up to three freeze-thaw cycles at -80°C. After protein precipitation, HCQ, DHCQ and DCQ in extract were stable at least 48 h in HPLC auto-sampler at 4°C. Long-term stability of HCQ and its major metabolites in whole blood at -80°C was not tested. In clinical laboratory, HCQ results have to be reported to patients within 5 days. The working standard in whole blood is always freshly made at the day of experiment.

### Blood collection

The Passing–Bablock linear regression analysis (n = 65) showed a good agreement between EDTA and lithium-heparinate tubes for assay of HCQ and its metabolites (Figure 2). Whatever the analyte, the Bland–Altman analysis did not show any significant bias between the two anticoagulants (data not shown). These results show for the first time that EDTA and lithium-heparinate tubes are both suitable for blood collection for HCQ TDM. Finally, the effect of the separator gel in lithium-heparinate tubes was low (<10%) on assay of HCQ, DCQ and DHCQ (Figure 3). The Bland–Altman analysis did not show any significant bias between tubes with gel separator and those without (data not shown). Taken together, these results suggest that there is no significant changes in whole blood concentration of HCQ and its metabolites while using lithium-heparinate blood collection tubes that contain a separator gel. The large diffusion of HCQ and its metabolites into the red blood cells can explain in part these results.

### Clinical application

The mean steady-state HCQ, DHCQ and DCQ concentration in 37 SLE patients (dose range: 200–600 mg/day) was 1336 ± 621 ng/ml, 1037 ± 721 ng/ml and 115 ± 54 ng/ml, respectively. For each compound, the mean steady-state concentration and the large inter individual variability in whole blood exposure (˜50%) are in accordance with those previously reported in the literature [12,14]. The Passing–Bablock linear regression analysis (n = 37) showed a good agreement between the current method and the Tett's method [15] with a correlation function: current method = 1.07 (95% CI: 0.97–1.20) × Tett's method + 152.9 (95% CI: 61.5–254.4) (Figure 4A). No significant deviation from linearity was observed (p > 0.1). The Bland–Altman analysis did not show any significant bias between the two methods (Figure 4B). In the same way, the Passing–Bablock linear regression analysis showed a good agreement between the current method and the LC–MS/MS method [20] with a correlation function: current method = 1.06 (95% CI: 0.97–1.16) × LC–MS/MS method + 126.8 (95% CI: 0.95–236.1) (Figure 5A). No significant deviation from linearity was observed (p > 0.1). The Bland–Altman analysis did not show any significant bias between the two methods (Figure 5B). Taken together, these results prove that the current method can be interchangeable with either Tett's method [15] or LC–MS/MS [20] method to assay whole blood HCQ concentrations in SLE patients.

## Conclusion & future perspective

A simple HPLC-FLD method with methanol and cupric sulfate protein precipitation has been validated to determine the concentration of HCQ and its metabolites in whole blood from SLE patients. The method was successfully implemented in routine clinical practice for the TDM of HCQ in SLE patients. Monitoring HCQ blood level can help prevent the emergence of disease flares and identify nonadherence. Poor treatment adherence has been related to poor disease control, increased morbility and mortality together with decreased quality of life. Nonadherence also results in a significant economic burden, such as hospitalization. This method may contribute to the spreading of HCQ monitoring, especially in hospital laboratories not having LC–MS/MS. The method may be used to monitor blood level of HCQ not only in SLE patients, but also in patients with sjogren's syndrome; lyme disease, arthritis; dermatomyositis. Finally, the interchangeability of the present method with the Tett's method [15] indicates that this new method could be used in the future clinical trials investigating the pharmacokinetic/pharmacodynamic relationship of HCQ in SLE patients.

**Table 1.** **Standard curves of hydroxychloroquine, desethylchloroquine and desethylhydroxychloroquine in human whole blood.**

**Standard**	**Linearity range (ng/ml)**	**Equation (n = 6)**	**r^2^**
HCQ	50–4000	Y = 12.3X	0.99945
DCQ	25–1000	Y = 41.2X	0.99940
DHCQ	25–2000	Y = 19.1X	0.99983

DCQ: Desethylchloroquine; DHCQ: Desethylhydroxychloroquine; HCQ: Hydroxychloroquine.

**Table 2.** **Precision and accuracy for hydroxychloroquine, desethylhydroxychloroquine and desethylchloroquine and in human whole blood.**

**Standard**	**Target concentration (ng/ml)**	**Intra-day (n = 10)**			**Inter-day (n = 20)**		
		**Mean observed concentration (ng/ml)**	**RSD (%)**	**Mean accuracy of target value (%)**	**Mean observed concentration (ng/ml)**	**RSD (%)**	**Mean accuracy of target value (%)**
HCQ	250	233	4.4	93.1	240	4.6	95.8
	1000	993	10.3	99.3	960	8.0	96.0
	2000	2020	4.3	101.0	1989	5.3	99.5
DHCQ	125	126	4.3	101.1	129	4.8	103.2
	500	511	10.3	102.2	502	8.4	100.4
	2000	1946	5.3	97.3	1962	1.9	98.1
DCQ	125	128	4.5	102.2	128	3.9	102.2
	500	495	10.3	99.0	493	8.8	98.6
	1000	1013	4.9	101.3	1026	4.6	102.6

DCQ: Desethylchloroquine; DHCQ: Desethylhydroxychloroquine; HCQ: Hydroxychloroquine; RSD: Relative standard deviation of the mean.

**Table 3.** **Recovery of hydroxychloroquine, desethylhydroxychloroquine and desethylchloroquine in human whole blood.**

**Standard**	**QC Sample (n = 3)**		
	**Concentration (ng/ml)**	**Recovery (%)**	**RSD (%)**
DHCQ	250	47.1	9.2
	1000	53.2	14.4
	2000	50.6	7.6
DCQ	250	52.0	10.4
	1000	57.7	15.0
	2000	55.2	7.7
HCQ	250	61.1	10.1
	1000	67.2	15.0
	2000	64.5	6.2
QN	5000	62.4	2.5

DCQ: Desethylchloroquine; DHCQ: Desethylhydroxychloroquine; HCQ: Hydroxychloroquine; QN: Quinine; RSD: Relative standard deviation of the mean.

**Executive summary**

The whole blood sample can be collected in systemic lupus erythematosus patients received treatment of hydroxychloroquine (HCQ).
Blood sample then be prepared by precipitating protein with methanol and cupric sulfate.
HCQ and its metabolites, desethylhydroxychloroquine and desethylchloroquine were separated on a HPLC phenyl column and monitored by fluorescence detection.
The method is very practical and was applied to routinely monitor the steady state whole blood exposure of HCQ and its metabolites in systemic lupus erythematosus patients. It well correlated with our LC–MS/MS and another HPLC method.
